# Supplementary figures and images for: Competition-function tradeoffs in ectomycorrhizal fungi
Source: PeerJ. 2016 Jul 27;4:e2270. doi: 10.7717/peerj.2270 (PMC4974999; doi:10.7717/peerj.2270)

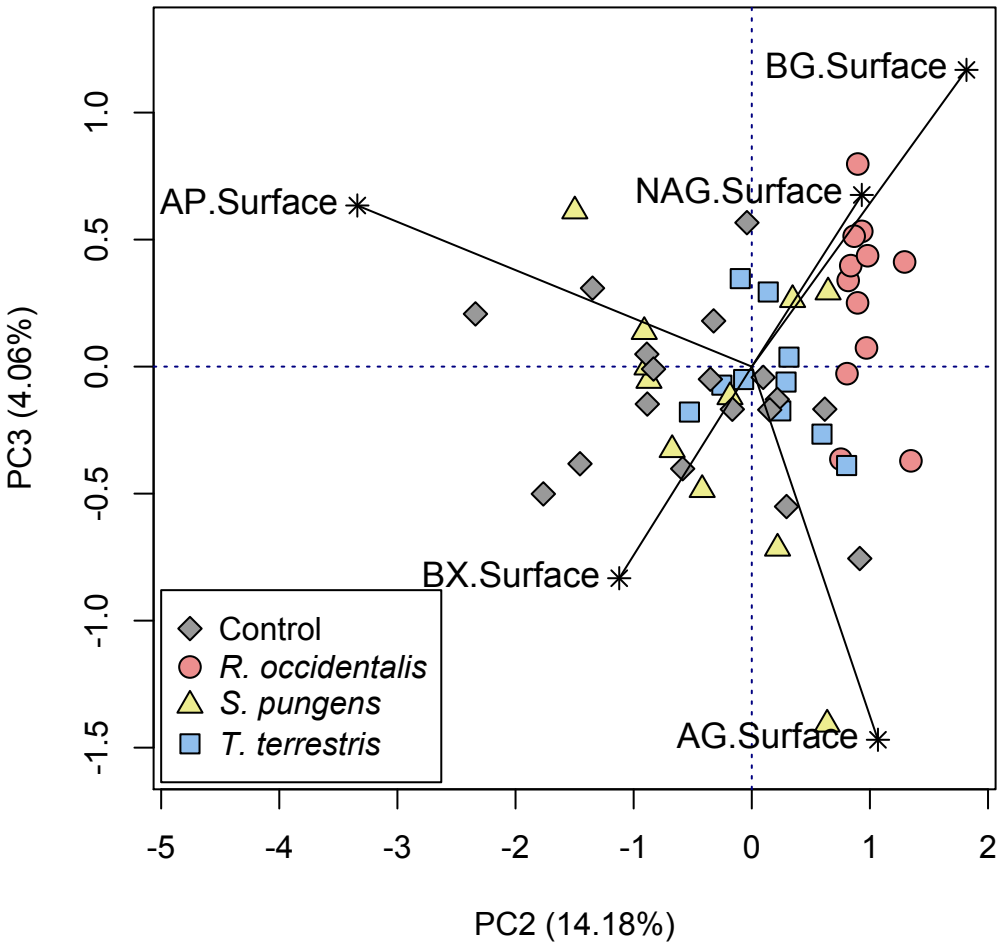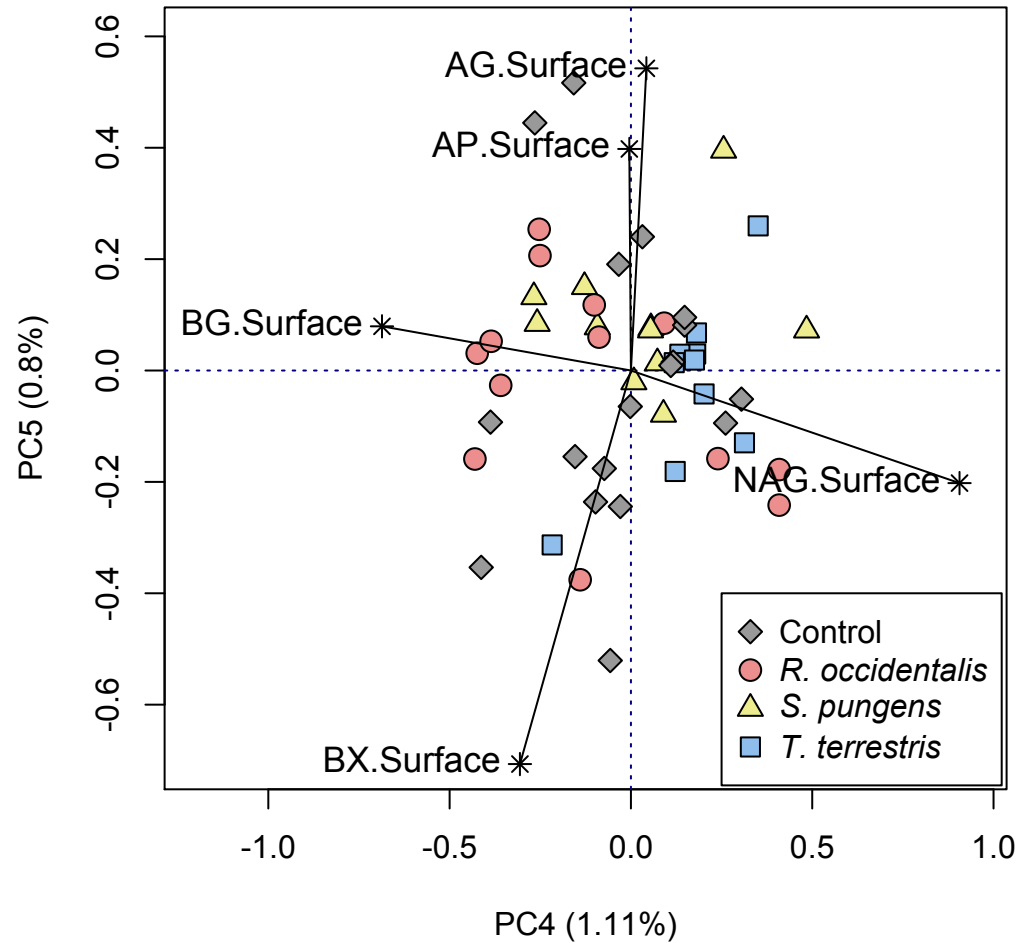

Supplement: Figure S1 — Axis loadings are given in Table 1. [file peerj-04-2270-s002.pdf]
